# Antifungal management in ICU: careful follow-up of voriconazole prescription needed!

**DOI:** 10.1186/s13054-023-04362-4

**Published:** 2023-02-23

**Authors:** Florian Reizine, Jean-Pierre Gangneux

**Affiliations:** 1 CH Vannes, Réanimation Polyvalente, 56000 Vannes, France; 2 CHU Rennes, Service de Parasitologie-Mycologie, 35033 Rennes, France; 3 Univ Rennes, CHU Rennes, Inserm, EHESP, IRSET (Institut de Recherche en Santé, Environnement et Travail) - UMR_S 1085, 35000 Rennes, France

Over the last 5 years, viral induced acute respiratory distress syndrome (ARDS) has emerged as a major risk factor for invasive fungal infections in ICU patients worldwide [1, 2]. Pulmonary aspergillosis, being reported in 15–27% of critical COVID-19 patients [2, 3] and in nearly 20% of Influenza associated ARDS patients [1], appears to be the main fungal infection involved. These frequent and deadly conditions have raised concerns regarding their management, with the increase use of targeted antifungal therapy in case of positive diagnosis. Moreover, as recently reported by Bienvenu et al., empirical antifungal therapy in COVID-19 patients hospitalized in ICU has been promoted in different centers while not consensual, but have resulted in an expected overconsumption of voriconazole [4, 5]. And regarding chemoprophylaxis of invasive pulmonary aspergillosis, posaconazole prescription has been investigated in viral ARDS patients [6, 7], but the impact of such early strategies on patient outcomes is still debated and yet to be evaluated.

Thus, the pandemic-induced increase in the consumption of antifungals warrants an optimal and multifaceted monitoring included in an antifungal stewardship program, especially for voriconazole therapy in ICU patients. Indeed, several aspects aside from consumption and expenditure should be considered to optimize the management of its use and limit adverse effects:

(i) First, the initiation of a targeted antifungal therapy must be considered in patients with possible, probable or proven invasive pulmonary aspergillosis as soon as the diagnosis is made. When an IPA is suspected, bronchoscopy with bronchoalveolar lavage remains the cornerstone of its diagnosis. The lack of robust data on both empirical or prophylactic antifungal treatments precludes any conclusions regarding these strategies. Treatment duration remains debated, a treatment course of 6–12 weeks has been suggested by an international consortium of experts [8]. However, while in immunocompromised patients longer treatment may be required, monitoring of the infection with biomarkers may allow treatment to be discontinued earlier (e.g. negative *Aspergillus* culture and/or PCR in BAL and negative galactomannan detection in BAL and/or in blood).
(ii) Second, voriconazole administration to ICU patients is highly challenging, as the non-linear pharmacokinetics of this drug can be considerably influenced by impaired renal or liver function, as well as by the use of extracorporeal membrane oxygenation [9] or renal replacement therapy. The clearance of antifungals can be very uncertain during intensive care unit hospitalization. Although increased renal clearance is common in ICU patients, it is difficult to identify patients at such risk of subsequent suboptimal voriconazole dosage. Moreover, altered volume of distribution (V_d_) is common in ICU patients. Endothelial dysfunction promoting expansion of the interstitial space along with hemodynamic management including intravenous fluid loading can result in the expansion of the V_d_ of these critical patients. In addition, the frequent occurrence of hypoalbuminemia in critical patients causing variation in plasma protein binding may also contribute to make the pharmacokinetics of antifungals unpredictable.
(iii) Third, changing from intravenous to the oral route should be promoted as soon as possible. However, digestive absorption may vary depending on the patient and clinical situations, and the enteral route can be contraindicated in cases of digestive dysfunction or recent surgery.
(iv) Fourth, the inflammatory state and potential drug-drug interactions in patients with severe COVID-19 may alter the metabolism of many drugs including voriconazole, making therapeutic drug monitoring (TDM) a necessity [10]. For instance, while analyzing clinical and therapeutic features of both influenza and COVID-19-associated pulmonary aspergillosis [11], we observed a significant delay to reach the optimal therapeutic range of voriconazole in CAPA patients that may have been favored by the use of corticosteroids [12]. This point emphasizes that TDM should be recommended for this particular population to ensure that therapeutic ranges are reached, in parallel with the promotion of empirical therapy. In addition, the development of pharmacological algorithms based on routine inflammatory biomarkers (e.g. C-Reactive Protein) deserves further investigations and could be considered to improve the management of patients treated by voriconazole [10].
(v) Fifth, traditionally, TDM has only been used to minimize the likelihood of toxicity for drugs with narrow therapeutic indices [13]. However, for severe COVID-19 patients who are particularly exposed to delirium [14], TDM of voriconazole could be considered as the cornerstone of the management of CAPA.
(vi) Finally, high antifungal consumption may contribute to an increased risk of the emergence of antifungal resistance, in addition to the risk of infection with a resistant environmental isolate [15]. Thus, the resistance of *Aspergillus* to triazole is a threat that could have a dramatic impact on the management of patients with invasive aspergillosis, and we believe that in vitro susceptibility should be monitored when evaluating antifungal strategies and considered as a pillar of an antifungal stewardship program.

In conclusion, the increased risk of invasive fungal infection in ICU patients is noteworthy and deserves an antifungal stewardship program to ensure appropriate choice, dose and duration of antifungal therapy along with indicators of good practices including antifungal consumption and expenditure, induced toxicity and the surveillance of drug-drug interactions through TDM, as well as in vitro susceptibility monitoring of *Aspergillus* strains.

## Data Availability

Not applicable.
